# The relationship between self-control and symptoms of anxiety and depression in patients with eating disorders: a cross-sectional study including exploratory longitudinal data

**DOI:** 10.1186/s40337-023-00750-x

**Published:** 2023-02-14

**Authors:** Emmi Eriksson, Mia Ramklint, Martina Wolf-Arehult, Martina Isaksson

**Affiliations:** 1 Department of Medical Sciences, Child and Adolescent Psychiatry, Uppsala University, Entrance 10, Floor 3B, 751 85 Uppsala, Sweden; 2 Department of Clinical Neuroscience, Centre for Psychiatry Research, Karolinska Institutet and Stockholm Health Care Services, Region Stockholm, Solna, Sweden

**Keywords:** Eating disorders, Personality style, Anxiety, Depression, Self-control, Overcontrol, Undercontrol

## Abstract

**Background:**

Personality style can partly be described as the way an individual controls and regulates emotions and can be divided into over- and undercontrol. Studies have indicated that personality style may impact the onset, clinical presentation, and recovery from an eating disorder (ED). Furthermore, symptoms of anxiety and depression are common in patients with EDs. However, the association between self-control levels and anxiety/depression symptoms in patients with EDs remains unknown. The main aim of this study was to assess how levels of self-control relate to anxiety/depression symptoms in patients with EDs, with a secondary, exploratory aim to assess the stability of self-control during treatment.

**Methods:**

Patients were recruited from the outpatient ED clinic at the Uppsala University Hospital, between October 2014 and December 2019. In total, 227 patients (age: 25.4, SD: 7.1) were included at the start of their treatment, with 14 participants also completing post-treatment measurements. Self-control was assessed with the Ego Undercontrol scale (EUC-13), anxiety/depression symptoms with the Hopkins Symptoms Checklist (HSCL-25), and ED diagnosis and symptoms with the Eating Disorder Examination Interview (EDE-I) and Questionnaire (EDE-Q), respectively.

**Results:**

A quadratic regression (n = 227) showed that levels of self-control accounted for about four percent of the variance in degree of global anxiety/depressive symptoms. Anxiety/depression symptoms were better explained by ED symptoms (R^2^ = 0.24). Visualizations in boxplots revealed a tendency for extreme values of both over- and undercontrol to be associated with higher levels of depression, whereas symptoms of anxiety increased with increasing undercontrol. In the exploratory analyses (n = 14) levels of self-control remained more stable than symptoms of anxiety and depression, which decreased significantly during ED treatment.

**Conclusions:**

Our results indicated that anxiety/depression symptoms, in patients with EDs, were not strongly correlated with levels of self-control, but rather with ED symptoms. However, extreme values of both over- and undercontrol showed a tendency to be associated with higher levels of depression symptoms, whereas anxiety symptoms increased with increasing levels of undercontrol. Future studies could benefit from considering both over- and undercontrol as potentially dysfunctional.

## Plain English summary

Eating disorders (EDs) are mental disorders characterized by a persevering behavioral disturbance in eating and distress in thoughts or emotions, significantly impairing psychosocial function and/or physical health. Anxiety and depression are common in patients with EDs.

Studies have indicated that personality style may impact the onset, clinical presentation, and recovery from an ED. Personality style can be studied in terms of self-control, ranging from over- to undercontrol. However, it remains unknown how self-control relates to anxiety/depression in patients with EDs and its stability during ED treatment. This is what we aimed to investigate in this study, collecting data before treatment in 227 adult patients from a Swedish outpatient ED clinic, with 14 also completing post-treatment measurements.

Our results indicated that global anxiety/depression levels in patients with EDs were not strongly correlated with levels of self-control, but rather with ED symptoms. Nevertheless, anxiety seemed to increase with increasing levels of undercontrol and extreme values of both over- and undercontrol showed a tendency to be associated with higher levels of depression. In the exploratory analyses self-control levels remained more stable during treatment than global anxiety/depression levels, which decreased significantly.

## Background

Eating disorders (ED) are mental disorders characterized by a persevering eating behavioral disturbance and associated distress in thoughts or emotions, significantly impairing psychosocial function and/or physical health [1]. Symptoms of anxiety and depression are common in patients with EDs [2]. Some studies indicate that up to 65% of the patients presenting with an ED also meet the criteria for an anxiety disorder [2], and depression affecting 46–74% of patients with anorexia nervosa (AN) and 30–60% of those with bulimia nervosa (BN) [3]. The expected lifetime incidence of an affective disorder in patients with EDs is up to 98% [4]. EDs are often severe disorders with an elevated mortality rate ratio of 2.87 [5], remission rates of approximately 45% after treatment, i.e., remaining symptoms for the majority of patients, and a relapse rate of 30% within the first year among the individuals in remission [6]. Thus, there is a need for improved understanding and treatment of these disorders.

Individual variations are often overlooked in patients with eating disorders, which presents challenges in establishing an acceptable, effective and feasible first‐line outpatient treatment [7]. A personality-oriented approach has been suggested as an alternative to established treatment or as a complementary way of optimizing treatment and predicting the clinical course and outcome in patients with EDs [7]. Several studies have identified three main personality styles among ED patients: an undercontrolled, an overcontrolled and a low psychopathology style, the latter also known as high-functioning perfectionistic or resilient [8–10]. These personality styles are related to the concept ego control, also known as self-control. Self-control refers to the ability to inhibit or express impulses to achieve long-term goals and can vary along a spectrum from undercontrol to overcontrol [11]. Undercontrol is characterized by impulsiveness, spontaneity, fluctuating emotions, and expressing these emotions even when socially inappropriate [11]. Overcontrol, on the other hand, can lead to overly organized behavior, as well as an ability to delay gratification and refrain from pleasure [11].

Self-control has previously been linked to ED treatment outcome, with undercontrolled patients having a higher risk of poor outcome at discharge [10]. However, it has also been shown that patients with an ED in addition to obsessive–compulsive personality disorder (OCPD) or autism spectrum disorder (ASD), diagnoses characterized by high levels of self-control or overcontrol, have poorer outcome compared with non-comorbid groups [12], hence suggesting that both overcontrol and undercontrol can be negative predictors for treatment outcome. Both over- and undercontrol have also been associated with more severe symptoms of mental disorders [13], including more severe ED symptoms [10, 14] compared to intermediate levels of self-control. Further, high levels of self-control are positively correlated with internalizing problems, such as depression and anxiety, whereas high levels of undercontrol have a positive correlation with externalizing problems [15], such as substance use disorder. Such externalizing problems are often associated with symptoms of anxiety and depression as secondary effects [16, 17]. However, the relationship between self-control and degree of anxiety and depression symptoms in the ED population has not been investigated. Understanding whether high and/or low levels of self-control are related to symptom severity of anxiety and depression symptoms could improve future possibilities of individualizing ED treatments, targeting not only ED symptoms but also anxiety and depression symptoms and personality style.

Personality is, by definition, a pattern that is stable over time, in both non-clinical and clinical groups [18]. However, there is increasing evidence that maladaptive personality characteristics may be treatable [19] and expanded knowledge about the stability of self-control levels might thus not only improve our understanding of the etiology and presentation of EDs, but also aid in developing more efficient treatments.

The main aim of this study was to evaluate whether levels of self-control relate to symptoms of anxiety and depression in patients with EDs, with a secondary, exploratory aim to study the stability of self-control levels during ED treatment. Considering the known association between over- and undercontrol to internalizing and externalizing problems, respectively, we hypothesized that ED patients with a higher degree of either over- or undercontrol would have more severe symptoms of anxiety and depression than patients with intermediate ratings. The secondary aim was purely exploratory.

## Methods

### Procedure and participants

Patients were recruited from the ED clinic at Uppsala University Hospital between October 2014 and December 2019. The ED clinic is an outpatient clinic for patients with AN and atypical anorexia nervosa (AAN), moderate to severe BN, and other complex ED cases with comorbidities or suicidality. Binge eating disorder (BED), avoidant restrictive food intake disorder (ARFID), and subclinical BN are generally not treated at the clinic, but generally referred to the obesity unit, primary or private care or the general psychiatry. All patients referred to the clinic, fulfilling the inclusion criteria of a minimum age of 18 years and an ED diagnosis were eligible for this study, and in total 227 patients were included at the start of their treatment. The inclusion was broad to represent a clinical sample. The exclusion criteria included being assessed as unable to participate and fill out questionnaires due to severe mental illness, i.e., when patients needed inpatient care or other emergency interventions, had insufficient knowledge of Swedish, or had limited cognitive abilities. ED diagnoses were determined by any one of 15 psychologists, trained in the Eating Disorder Examination Interview (EDE-I) by one expert. To assess the inter-rater reliability six filmed interviews conducted by the expert, were assessed by all the 15 trained psychologists, who showed complete agreement in all ratings. As the University hospital, where the study was performed, used DSM-IV for diagnostics when the study was initiated, a decision was made to continue diagnosing according to the DSM-IV throughout. Afterwards, a recoding in accordance with the DSM-5 criteria was performed so that the sample could be presented in accordance with the revised diagnostic criteria. The group of other eating disorders included ARFID (n = 2), other specified eating disorders (OSFED): BN of low frequency (n = 16), purging disorder (n = 14), and others (n = 21), where the reason for not fulfilling a specific ED diagnosis was another than exemplified OSFED groups in the DSM-5. BED (n = 6) was also included in the group of other eating disorders, since it was rarely treated at the clinic.

All patients who decided to participate in the study received verbal and written information about the study at their first meeting during the psychological assessment phase and gave written informed consent. Weight and height measurements were performed at the clinic and background variables such as age, gender, marital status, occupation and level of education were collected at baseline.

**Fig. 1:**
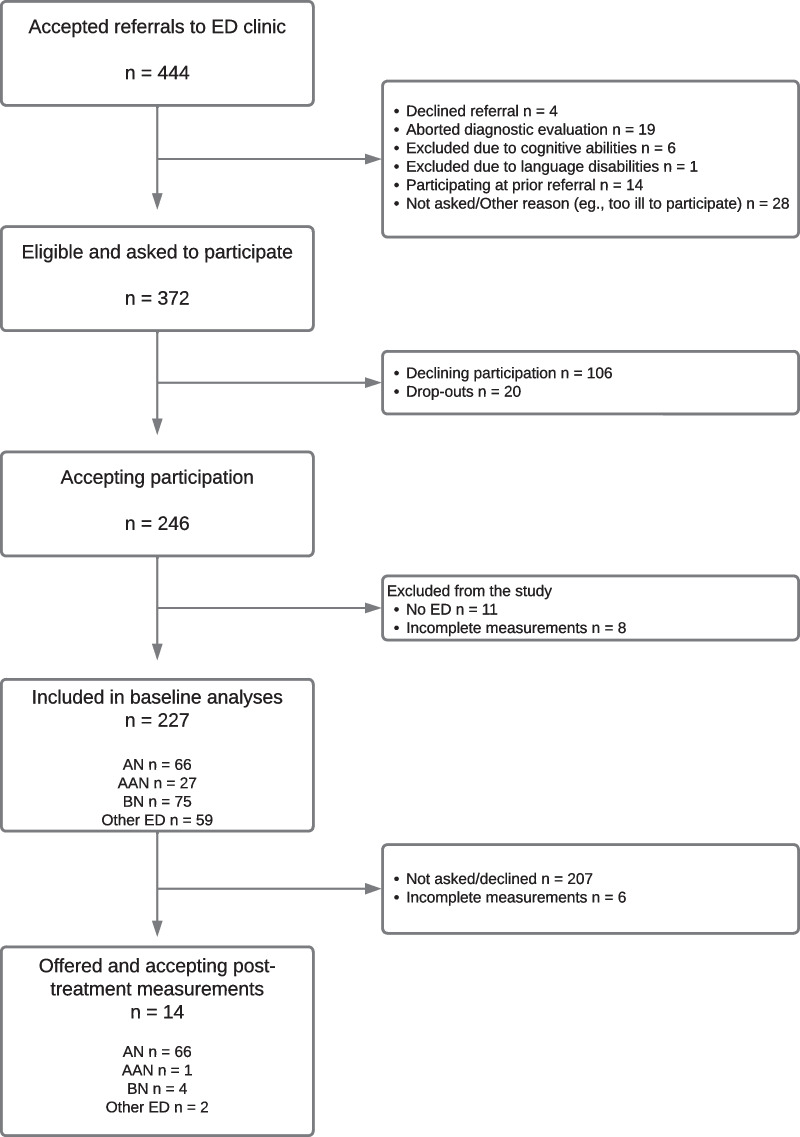
Flowchart of participant recruitment. AAN = Atypical Anorexia Nervosa**.** AN = Anorexia Nervosa**.** BN = Bulimia Nervosa**.** ED = Eating Disorder

At the time of their last therapy session, a subset of patients were asked to fill out questionnaires yet again. Twenty agreed, with 14 patients completing all questionnaires, i.e., EDE-Q, HSCL-25 and EUC-13 at both time-points. Due to organizational issues at the clinic, the post-treatment measurements were not always handed out and collected as planned, explaining the small sample. The 14 patients had received Enhanced Cognitive Behavioral Therapy, typically consisting of 20 sessions for BN and 40 sessions for AN patients, over the same number of weeks. 10 had reached ratings within one SD of the mean of a non-clinical sample, indicating possible remission [Eating Disorder Examination Questionnaire (EDE-Q) ≤ 2.83]. The recruitment process is shown in Fig. 1.

### Instruments

#### EDE-I and EDE-Q

All participants underwent the semi-structured EDE-I at baseline, to determine ED diagnosis. The interview was conducted by a trained psychologist and focused on attitudes and behavior central to ED pathology [20].

The EDE-Q is a well-established self-report instrument with 36 items, derived from the EDE-I, to assess ED symptoms. The attitudinal items are divided into four subscales—restraint, eating concern, shape concern, and weight concern—and are rated by patients for severity or frequency, using a 7-point rating scale. Responses to behavioral questions, for instance on binge eating and vomit induction, are given as number of days. The level of ED psychopathology increases with a higher score. As with the EDE-I, the questions cover the preceding 28 days. Swedish norms are available with a mean score of 1.56 (SD = 1.27), with 84% of the population scoring 2.83 or below, in the non-clinical population [21]. Considering the prevalence of EDs, scores below this value can be used as an indication of EDE-Q values similar to those in a non-clinical population and, in the absence of other measures, as a proxy for remission or at least partial remission. Correlations with other measures of similar constructs range from 0.40 to 0.79 for the different subscales [22].

Data support the test–retest reliability of both EDE-I and EDE-Q, as well as their validity in differentiating those with ED pathology from non-clinical controls. The internal consistency of EDE-Q is acceptable to high, with the different subscales ranging from coefficient alpha 0.7 to 0.93 [22].

#### EUC-13

The Ego Undercontrol Scale—short version (EUC-13) is a self-rating questionnaire that contains 13 items, measuring levels of expression and inhibition of emotional impulses on a single spectrum ranging from over- to undercontrol. The EUC-13 is based on a three-factor model consisting of the subscales uninhibited behavior (five items), planful conscientious behavior (five items), and socially restrained behavior (three items). The items on each subscale are rated on a four-point Likert scale, ranging from “Disagree very strongly” [1] to “Agree very strongly” [4]. When calculating the final score, the subscales of planful conscientious behavior and socially restrained behavior are reversed. Thus, a high score indicates low levels of emotional impulse inhibition (high undercontrol). The EUC-13 has been validated in a non-clinical Swedish community sample, showing adequate test–retest reliability, in addition to construct validity and acceptable internal consistency (α = 0.71) [23]. However, use of the global score is recommended due to its higher validity [24].

#### HSCL-25

The Hopkins Symptom Checklist-25 (HSCL-25) is a 25-item self-report questionnaire designed to screen for anxiety (10 items) and depression (15 items) symptoms. The respondent rates each symptom, depending on how bothersome it has been during the week prior to assessment, on a scale ranging from “Not at all” [1] to “Extremely” [4, 25]. The scale has been widely used for screening, with a cut-off of 1.75, demonstrating accuracy when validated against various diagnostic interviews worldwide and has a high internal consistency, with a coefficient alpha of 0.80 for both subscales, respectively [26]. The scale’s median average score has also shown high correlation with other measures of mental health [27], and satisfactory validity and reliability in assessing mental symptoms [28]. The scale is validated in a Swedish sample [26].

### Data collection and analyses

Missing values for single questions in EUC-13 and HSCL-25 were handled through single imputation using the mean of the affected subscale, as long as the missing values were limited to less than 20% of the subscale, in accordance with recommendations [29]. If this limit was exceeded, the entire scale was omitted for that individual. However, missing values were rare (< 0.5%). For the EDE-Q, a global score was calculated as the mean score of all rated items, as long as at least 50% of the items were rated, in accordance with the scoring recommendations [30].

Normal distribution of the data was checked by visualization in histograms, and through the values of skewness and kurtosis, and data were further checked for outliers using box- and scatterplots.

The relationship between global HSCL-25 scores and the EUC-13 was initially visualized in a scatterplot and through locally estimated scatterplot smoothing (LOESS), which indicated a slight curvilinear trend. An analysis of variance (ANOVA) was conducted, further strengthening indications of a quadratic term, with a higher R^2^ and a lower standard error of the estimate, in addition to a significant F-ratio and nonsignificant Levene’s test. Additionally, a linear regression was tested, resulting in similar, but somewhat lower, explained variance than in the quadratic regression. A quadratic regression model was therefore chosen, with global HSCL-25 score as the dependent variable, as well as a model with the subscale for depressive symptoms as the dependent variable. As the LOESS of levels of self-control in relation to symptoms of anxiety showed a linear trend, a linear regression was chosen for this analysis. Assumptions for these models were tested, all of which showed the data as suitable for regression models. The analysis was also conducted excluding the rarer and atypical eating disorders OSFED: ARFID, purging disorder, and OSFED: others with essentially unchanged results, and these participants were therefore included in all analyses.

Next, a visual representation of potential confounders was performed in a directed acyclic graph. Adjustments for gender, age, and starvation were found to be necessary to estimate the effect of EUC-13 scores on HSCL-25 scoring, for which we applied BMI as a proxy. The potential confounding effect of EDE-Q was more unclear, depending on whether the relationship between EUC-13 and EDE-Q scoring was considered uni- or bidirectional. Subsequently, two adjusted models were created, one correcting only for gender, age, and BMI, and the other also including EDE-Q scores. Correlations between the described factors and the outcome, HSCL-25, were tested, and the factors were checked for data multicollinearity prior to analyses, showing weak to non-significant correlations. Data on confounding factors were available for 220 of the participants.

To examine how extreme values of self-control, i.e., over- and undercontrol, related to the degree of symptoms of anxiety and depression, measured with HSCL-25, the participants were divided into four groups, based on their EUC-13 scores, for a subset of analyses. These groups were defined by standard deviation (SD) from the sample mean, in steps of 1 SD. The number of groups was chosen to enable comparison between what may be considered functional levels of over- and undercontrol (in the groups − 1 and + 1 SD from the sample mean, respectively) and those with more extreme levels, while still ensuring sufficient numbers of participants in each group. This generated the following groups: highly overcontrolled (n = 32), overcontrolled (n = 101), undercontrolled (n = 68), and highly undercontrolled (n = 26). The analyses conducted in these groups included visualization in boxplots for the two subscales anxiety and depression symptoms, a one-way ANOVA, and Tukey’s test.

For the exploratory analyses in the subgroup of participants with both pre- and post-treatment measurements, sample differences were tested with Wilcoxon’s signed-rank test, which was chosen due to the small sample size. Data was further visualized in side-by-side boxplots and the reliable change index was calculated for each individual.

Analyses were all conducted with SPSS Statistics version 28.0.1.0.

## Results

Participant demographics are presented in Table 1.

**Table 1:** Descriptive data on participants (n = 227) at baseline

*Gender*	
Woman	216 (95.2%)
Man	9 (4.0%)
Other	2 (0.9%)
Mean age, years (SD) (Range)	25.4 (7.1) (18–59)
Mean BMI, kg/m^2^ (SD)^a^ (Range)	21.4 (4.8) (15.0–40.4)
*DSM 5 diagnosis*	
AN	66 (29%)
AAN	27 (11.9%)
BN	75 (33.0%)
Other ED	59 (25.9%)
*Level of education*^*b*^	
Elementary school	30 (13.2%)
High school	113 (49.8%)
Higher education	82 (36.1%)
*Main occupation*^*c*^	
Paid work	78 (34.4%)
Student	102 (44.9%)
Sick leave	38 (16.7%)
Unemployed	3 (1.3%)
Other	4 (1.8%)
*Marital status*^*d*^	
Single	131 (57.7%)
Married/Relationship	79 (34.8%)
Other	15 (6.6%)

*AAN* Atypical Anorexia Nervosa, *AN* Anorexia Nervosa¸ BN Bulimia Nervosa, *ED* Eating Disorder, *SD* Standard deviation

^a^3 patients missing data on BMI

^b^1 missing level of education

^c^2 missing main occupation

^d^2 missing marital status

### EUC-13 and HSCL-25

**Fig. 2:**
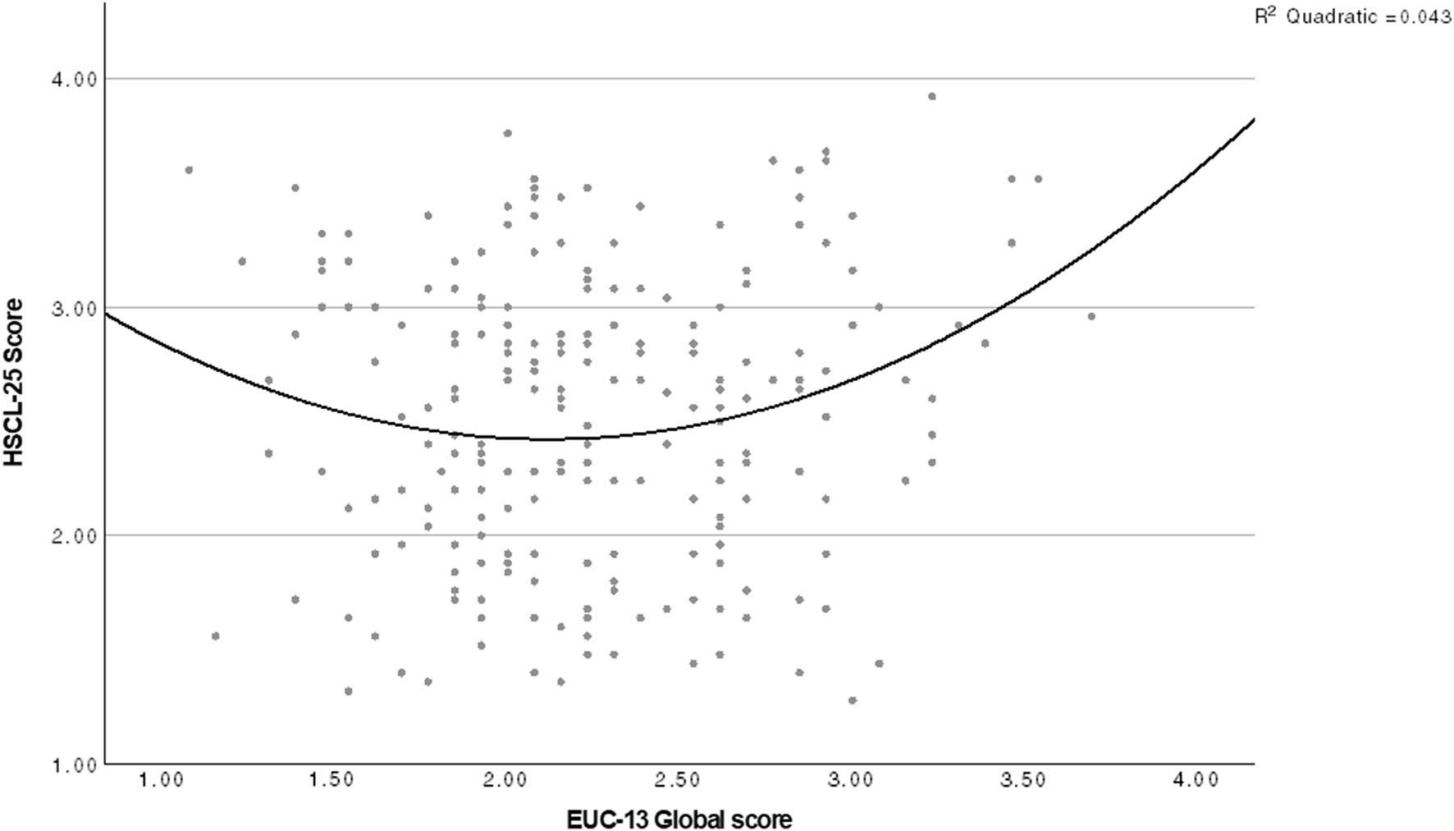
Scatterplot and quadratic model of global anxiety/depression symptoms in relation to self-control levels. Anxiety/depression symptoms are measured with Hopkins symptom checklist 25 (HSCL-25) and levels of self-control with Ego Undercontrol scale 13 (EUC-13) in a group of patients with an eating disorder diagnosis (n = 227), pre-treatment

The quadratic regression showed a statistically significant trend that patients exhibiting over- and undercontrol on the EUC-13 tended to score higher on global anxiety/depression symptoms, measured using HSCL-25 [F (2, 224) = 4.98, *p* = 0.008]. The EUC-13 was a statistically significant coefficient (*p* = 0.009) in the model. However, the proportion of variance explained by the EUC-13 was low (R^2^ = 0.043): four percent of the variance in anxiety/depression symptoms was explained by self-control. The graph for this is presented in Fig. 2. When running the same analysis for the subscale covering depression, and a linear regression for anxiety symptoms, the explained variance was essentially the same (R^2^ = 0.03 for both depression and anxiety symptoms), with both over- and undercontrol being associated with depression and anxiety symptoms increasing with increasing levels of undercontrol.

The results remained stable, and EUC-13 remained a statistically significant coefficient (*p* = 0.034) in the model, when adjustments were made for gender, age and BMI (R^2^ = 0.078, *p* = 0.007). However, when the analysis was conducted controlling for gender, age, BMI, and EDE-Q scores, the effect of the EUC-13 scores was non-significant (*p* = 0.09), with EDE-Q scores being the strongest predictor of global levels of anxiety/depression symptoms (R^2^ = 0.24, *p* < 0.001).

**Fig. 3:**
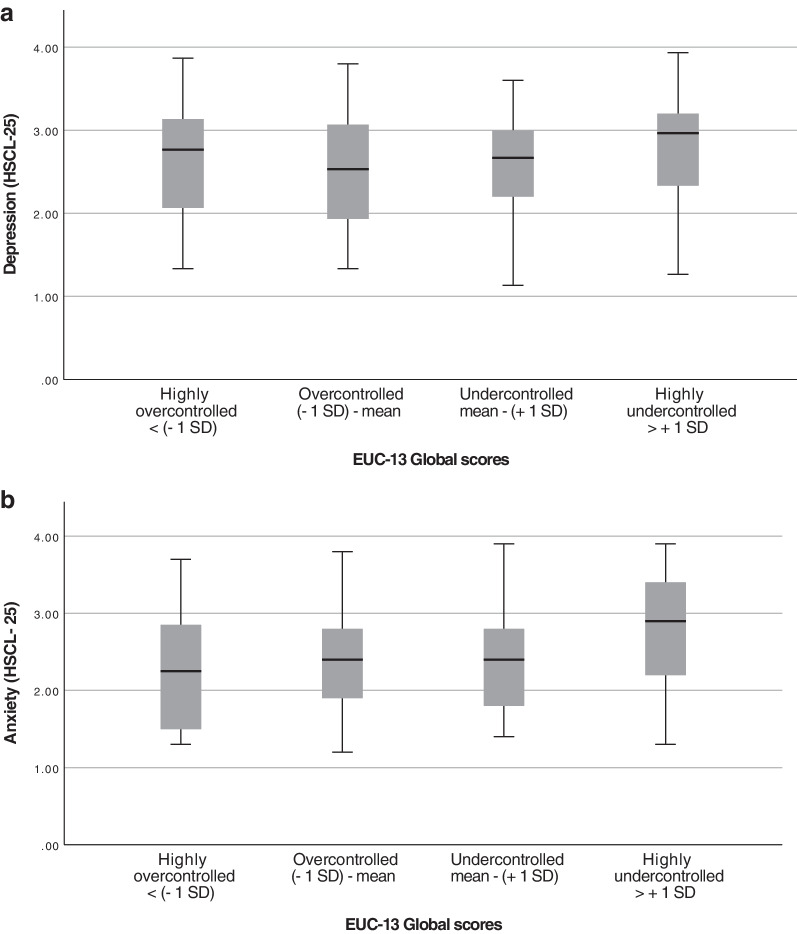
Boxplots of symptoms of depression (top) and anxiety symptoms (bottom) in relation to self-control levels. Symptoms of depression and anxiety are assessed with Hopkins symptom checklist 25 (HSCL-25) and the degree of self-control is measured with Ego undercontrol scale 13 (EUC-13), in a group of patients with an eating disorder, pre treatment. The results are presented in four groups with different levels of self-control, based on the standard deviation from the sample EUC-13 mean score (n = 227): highly overcontrolled (n = 32), overcontrolled (n = 101), undercontrolled (n = 68), and highly undercontrolled (n = 26)

Visualizations of the HSCL-25 subscales anxiety and depressive symptoms in boxplots for the four EUC-13 groups are presented in Fig. 3. In the ANOVA, the difference between groups for the subscale covering anxiety symptoms was statistically significant (*p* = 0.040, η2 = 0.036) though that for depressive symptoms was not (*p* = 0.30, η2 = 0.016). A follow-up Tukey’s test showed that the difference in anxiety levels between groups stemmed from the difference between the highly over- and highly undercontrolled groups (*p* = 0.044). The distribution between different ED diagnosis in the different groups of self-control levels, based on EUC-13, is presented in Table 2.

**Table 2:** Distribution of eating disorder diagnosis in different groups of self-control levels

Diagnosis	Highly overcontrolled < (− 1 SD)	Overcontrolled (− 1 SD) − Mean	Undercontrolled Mean – (+ 1 SD)	Highly undercontrolled > + 1 SD
AN	13 (40.6%)	34 (33.7%)	15 (22%)	4 (15.4%)
AAN	5 (15.6%)	16 (15.8%)	5 (7.3%)	1 (3.8%)
BN	8 (25%)	23 (22.8%)	27 (39.7%)	17 (65.4%)
Other ED	6 (18.8%)	28 (27.78%)	21 (30.9%)	4 (15.4%)

Self-control levels are measured with Ego undercontrol scale 13 (EUC-13), in a group of patients with an eating disorder, pre-treatment. The groups are based on the standard deviation from the sample EUC-13 mean score (n = 227) and consist of highly overcontrolled (n = 32), overcontrolled (n = 101), undercontrolled (n = 68), and highly undercontrolled (n = 26)

*AAN* Atypical Anorexia Nervosa, *AN* Anorexia Nervosa, *BN* Bulimia Nervosa, *ED* Eating Disorder

### EUC-13 and HSCL-25 pre- and post-ED treatment: exploratory analyses

The mean pre-treatment EUC-13 value was 2.36 (SD = 0.60), decreasing to 2.21 (0.40) post-treatment (n = 14). A Wilcoxon’s signed-rank test showed that this difference in the mean was non-significant (*p* = 0.17). However, the within-group variance decreased from 0.36 pre-treatment to 0.16 post treatment, and from 0.40 to 0.09 in the subgroup of patients who had achieved possible remission (n = 10). Among these nine cases, four showed reliable changes, all towards the mean, whereas five remained unchanged or with non-reliable changes. For HSCL-25, the global mean pre-treatment score was 2.67 (SD = 0.69), with a significant decrease to 1.96 (0.51) post-treatment (Wilcoxon, *p* = 0.004). Individual mean differences pre- and post-treatment in both EUC-13 and HSCL-25, and the calculated reliable change index (RCI) are presented in Table 3.

**Table 3:** Individual mean self-control levels and depression/anxiety symptoms, pre- and post-ED treatment (n = 14)

ID	EUC-13 pre	EUC-13 post	EUC-13 mean difference	EUC-13 RCI	EUC-13 direction of change	HSCL-25 global pre	HSCL-25 global post	HSCL-25 global mean difference	HSCL-25 global RCI	HSCL-25 global direction of change
1^A^	1.54	2.54	1	7.4	**Towards mean**	3.20	2.32	− 0.88	− 4.2	**Decrease**
2^A^	2.23	2.08	− 0.15	− 1.1	Towards extremes	3.08	1.64	− 1.44	− 6.9	**Decrease**
3^A^	3.46	2.31	− 1.15	− 8.5	**Towards mean**	3.28	1.72	− 1.56	− 7.5	**Decrease**
4^A^	2.77	2.23	− 0.54	− 4.0	**Towards mean**	3.64	2.40	− 1.23	− 5.9	**Decrease**
5^A^	2.23	2.08	− 0.15	− 1.1	Towards extremes	2.84	2.32	− 0.52	− 2.5	**Decrease**
6^A^	2.00	2.15	0.15	1.1	Towards means	2.12	1.24	− 0.88	− 4.2	**Decrease**
7^A^	2.23	2.23	0	0	No change	2.32	1.40	− 0.92	− 4.4	**Decrease**
8^A^	2.62	2.46	− 0.16	− 1.2	Towards mean	2.24	1.56	− 0.68	− 3.2	**Decrease**
9^A^	1.54	2.08	0.54	4.0	**Towards mean**	1.32	1.24	− 0.08	− 0.4	Decrease
10^A^	3.38	3.08	− 0.3	− 2.2	**Towards mean**	2.84	2.12	− 0.72	− 3.4	**Decrease**
11	2.85	2.38	− 0.47	− 3.5	**Towards mean**	3.36	2.92	− 0.44	− 2.1	**Decrease**
12	1.85	1.54	− 0.31	− 2.3	**Towards extremes**	3.20	2.24	− 0.96	− 4.6	**Decrease**
13	2.38	2.31	− 0.07	− 0.5	Towards mean	2.24	1.84	− 0.40	− 1.9	Decrease
14	1.92	1.46	− 0.46	− 3.4	**Towards extremes**	1.64	2.48	0.84	4	**Increase**

Self-control levels are measured with Ego Undercontrol scale (EUC-13) and symptoms of anxiety and depression with Hopkins Symptom Checklist 25 (HSCL-25), in 14 participants with an eating disorder diagnosis. The table further shows calculated differences in mean values, reliable change index (RCI) and direction of change

Directions of change for the EUC-13 scores are presented as towards initial sample mean (2.24) or towards the extremes. For the HSCL-25 scores, the direction of change is presented as decrease or increase. Reliable changes are shown in bold

^A^Having reached EDE-Q values within one SD of a non-clinical mean (participants 1 – 10)

## Discussion

The main aim of this study was to evaluate whether levels of self-control relate to symptoms of anxiety and depression in patients with EDs, with a secondary, exploratory, aim to study the stability of self-control levels during ED treatment.

The main finding was that the levels of anxiety and depression symptoms in patients with EDs were not strongly correlated with levels of self-control, but rather with ED symptoms. However, there was a tendency for extreme values of both over- and undercontrol to be associated with higher global levels of anxiety/depression symptoms, supporting our initial hypothesis. When the subscales were studied separately, symptoms of anxiety seemed to increase with increasing levels of undercontrol, whereas depressive symptoms were somewhat—though not statistically significantly—higher in both the most over- and the most undercontrolled. In our exploratory analyses, self-control levels seemed to remain more stable at the post-treatment assessment than the level of symptoms of anxiety and depression, which decreased significantly.

The proportion of global anxiety/depression symptoms explained by levels of self-control was low (about 4%), but statistically significant. These results were stable when investigating the impact of the confounding factors of gender, age, and BMI. However, when correcting for EDE-Q scores, the impact of self-control levels lost significance. It is therefore possible that other factors, such as the degree of ED symptoms, are of greater importance than personality style for predicting levels of anxiety and depression symptoms. Nevertheless, the impact of the EDE-Q as a confounder is questionable, as extremely high or low levels of self-control may increase ED symptoms, which in turn would increase levels of anxiety and depression symptoms. Thus, ED symptoms may form part of the causal pathway of self-control generating higher global levels of anxiety/depression symptoms; adjusting for the EDE-Q may therefore lead to overcorrection [31]. It can be argued that the model adjusted only for gender, age, and BMI was the most appropriate one. Additionally, the low levels of explained variance in our model may, at least partly, be explained by the scarcity of participants exhibiting pronounced levels of over- and undercontrol, giving them less impact in the model.

When the subscales anxiety and depressive symptoms were studied separately, the degree of anxiety symptoms seemed to increase with increasing levels of undercontrol, whereas levels of depression symptoms showed tendencies of a curvilinear trend with both over- and undercontrol being associated with higher levels of depressive symptoms. However, the effect sizes between groups of different levels of self-control were small.

Our findings add some evidence to the conceptualization of self-control as a spectrum, with both strong under- and overcontrol being associated with higher degrees of internalizing symptoms such as anxiety/depression symptoms. This is in contrast to the views of some theorists who consider only undercontrol to be disadvantageous [10]. Instead, our findings are in line with earlier studies associating both high levels of over- and undercontrol with more severe symptoms in patients with mental disorders [13]. However, the fact that the relationship between the subscales anxiety and depressive symptoms and self-control levels differ may indicate that the mechanism behind these symptoms varies between the overcontrolled and undercontrolled groups. Previous studies have linked overcontrol to social isolation [32, 33] and it has been suggested that depressive symptoms could be a consequence of such isolation. Undercontrol, on the other hand, is associated with impulsiveness, aggression, and more frequent conflicts [34], which may be crucial factors generating higher levels of anxiety and depression symptoms. The idea that the personality styles may cause different behavioral, emotional, and attitudinal problems raises the question of whether it could be beneficial to individualize therapy based on levels of self-control.

Further, differences in the distribution between type of ED diagnosis could be seen in the groups of different self-control levels. AN predominated in the highly overcontrolled group, and BN in the highly undercontrolled group, in line with previous studies [35, 36].

In the exploratory measurements before and after ED treatments, the EUC-13 mean value remained stable, and the majority of the studied individuals showed no significant tendency of normalizing levels of self-control. However, since the within-group variance decreased after treatment, the frequency of over- and undercontrol was reduced. This tendency was more pronounced in the subsample that achieved possible remission, somewhat contrasting with previous studies of the stability of personality in psychiatric populations [18, 37, 38]. Instead, it aligns with the belief that changes in personality style are, to some extent, a complication of an ED, or a so-called state effect, normalizing with decreasing ED pathology and starvation [10] and previous studies suggesting a change in personality in the direction of healthy controls, induced by recovery [39–41]. However, our exploratory results merely indicate a trend towards normalization of extreme levels of self-control, not a transition from over- to undercontrol, which could still be in line with the theory of a stable personality style. In addition, the majority of both the entire group and the subsample achieving possible remission exhibited no reliable changes, and the levels of self-control remained more unchanged than the degree of global anxiety and depression symptoms, which decreased significantly at post-treatment. However, due to the small sample size (n = 14), no conclusions could be drawn from these sub-analyses; the results must instead be considered exploratory.

It might be speculated that patients exhibiting a tendency to normalize levels of self-control during ED treatment or weight restoration, would be unlikely to benefit from interventions based on personality style, assuming that the initial extreme self-control levels were primarily a consequence of starvation, subsiding with weight restoration. However, patients with unchanged self-control levels despite remission or with remaining high levels of ED symptoms could potentially benefit from interventions directed at maladaptive self-control levels. This as both pronounced over- and undercontrol have been shown to correlate with low mental health, potentially increasing the risk for relapse in an ED or another diagnosis frequently associated with maladaptive levels of self-control, such as OCPD [42] or substance abuse [43]. This is hypothetical and not evaluated in this study, and thus requires further research.

### Limitations and strengths

This study has several limitations. First, there were several factors influencing levels of anxiety and depression symptoms that we were not able to control for in our analyses, such as traumatic life events, comorbidities, and current medication with for example antidepressants or anxiolytics. Secondly, there are large gender group differences, inhibiting any analyses on gender differences. Furthermore, when controlling for starvation, we used BMI as a proxy, and although BMI can serve as an indication of starvation over time, it is not ideal for assessing current caloric intake.

Additionally, due to the study’s cross-sectional design, no conclusions of causality could be drawn from our results. Also, it is important to remember that the EUC-13 cannot distinguish between functional and dysfunctional levels of self-control, but rather measures personality style without considering functional impairments. When dividing the data in categories based on sample mean and standard deviations, the categories become somewhat arbitrary, which may be problematic. Nevertheless, the aim of the study was to examine the highest and lowest levels of over- and undercontrol, with an increased risk of a dysfunctional level, and a division by standard deviation made this possible, making it appear to be an appropriate choice. Further, the outlying groups were defined by the deviation of more than one SD from the mean, which, it may be argued, is not extremely outlying. This division was made to ensure sufficient numbers of individuals in all groups. However, future studies should aim to include an even bigger sample of participants, enabling analyses of more outlying self-control levels.

An obvious limitation to the post-treatment analyses is the small study sample of 14 participants, a result of organizational issues that made data collection difficult. Offering compensation for participation, sending out reminder emails, and offering digital questionnaires could have increased participation rates and should be considered in future studies.

The ecological validity of this study is high, since the study was conducted in a clinical sample. However, patients were excluded when they required inpatient care, at least until their condition stabilized somewhat, which means that the generalizability of the study is limited in those with the most severe illness.

The main strength of this study is its number of participants, which was larger than commonly seen when investigating personality style in ED populations. Furthermore, the measures used—EUC-13, HSCL-25 and EDE-Q—are all previously validated in Swedish samples.

### Conclusion and future research

Our results indicate that the levels of anxiety/depression symptoms in ED patients are not strongly correlated with levels of self-control, but rather with ED symptoms. Nevertheless, there is a tendency that extreme values of both over- and undercontrol are associated with higher levels of depression, whereas anxiety symptoms seem to increase with increasing levels of undercontrol. Future studies could benefit from considering both over- and undercontrol as potentially dysfunctional. In the exploratory analyses self-control levels seemed to move towards normalization after ED treatment for a subset of participants, though levels of anxiety and depression symptoms decreased to a higher degree. Future research should aim to assess the stability of self-control levels post-ED treatment by studying a larger sample. Future studies should also aim to more closely examine potential differences between over- and undercontrolled patients, as well as differentiating between functional and dysfunctional under- and overcontrol and potential gender group differences. A broadened understanding of this field could aid development of more individualized and efficient ED treatments.

## Data Availability

Data will not be made publicly available due to confidentiality but can be provided upon reasonable request to the corresponding author.

## Abbreviations

AAN: Atypical anorexia nervosa
AN: Anorexia nervosa
ANOVA: Analysis of variance
ARFID: Avoidant/restrictive food intake disorder
ASD: Autism spectrum disorder
BED: Binge eating disorder
BN: Bulimia nervosa
BMI: Body mass index
ED: Eating disorder
EDE-I: Eating disorder examination interview
EDE-Q: Eating disorder examination questionnaire
DSM-5: Diagnostic and statistical manual of mental disorders, 5th edition
DSM-IV: Diagnostic and statistical manual of mental disorders, 4th edition
EUC-13: Ego undercontrol scale 13
HSCL-25: Hopkins symptom checklist 25
LOESS: Locally estimated scatterplot smoothing
OSFED: Other specified eating disorders
OCPD: Obsessive–compulsive personality disorder
RCI: Reliable change index
SD: Standard deviation
